# A Narrative Review of the Association between Obstructive Sleep Apnea and Glaucoma in Adults

**DOI:** 10.3390/ijms231710080

**Published:** 2022-09-03

**Authors:** Barbara Leggewie, Haralampos Gouveris, Katharina Bahr

**Affiliations:** 1 Department of Otorhinolaryngology, Head and Neck Surgery, University Hospital Bonn, 53127 Bonn, Germany; 2 Department of Otolaryngology, Head and Neck Surgery, University Medical Center Mainz, 55131 Mainz, Germany

**Keywords:** obstructive sleep apnea, glaucoma, sleep-disordered breathing, RNFL, AHI

## Abstract

Background: Obstructive sleep apnea (OSA) is a sleep disorder, primarily of the upper airway, which not only has a significant impact on quality of life but is also associated with various systemic diseases. Several ophthalmological diseases are also associated with OSA, especially glaucoma. The purpose of this review is to take a closer look at the causality and mutual influence. Methods: A systematic literature search was conducted using PubMed. A total of 19 studies with 316,178 adult participants were included. Results: Eleven of the sixteen studies concentrating on the prevalence of glaucoma in patients with OSA showed an association of both entities. One paper found a higher risk for progression of glaucoma in OSA patients. Five of the sixteen included studies failed to show a correlation between OSA and glaucoma. One study out of three surveying specific ophthalmological parameters showed an influence of OSA therapy on retinal nerve fiber layer (RNFL) thinning and vision. One study showed a rise in intraocular pressure (IOP), while two other studies showed no increase under continuous positive airway pressure (CPAP). Conclusions: Our findings suggest an association between OSA and glaucoma and, especially, between OSA and thinning of RNFL. CPAP therapy appears to be also suitable for patients with comorbid glaucoma.

## 1. Introduction

OSA is a chronic condition characterized by phases of partial or even complete upper airway obstruction during sleep, usually accompanied by snoring. These episodes lead to oxygen desaturation, hypercapnia and recurrent arousals with concomitant activation of the sympathetic nervous system, resulting in sleep fragmentation [1,2,3]. The severity of respiratory distress in OSA is described by the Apnea–Hypopnea Index (AHI), which measures the total number of apneas and hypopneas per hour: an AHI < 5/h indicates no OSA, AHI of 5/h to 15/h indicates a mild OSA, AHI of 15/h to 30/h indicates a moderate OSA and AHI > 30/h indicates a severe OSA [4]. Polysomnography is currently the gold standard for sleep monitoring. Apart from snoring, excessive daytime sleepiness, attention deficits, morning headaches and impaired daytime performance are frequent symptoms of OSA. Besides the mentioned effects on sleep quality, arousals also lead to critical increases in blood pressure, oxidative stress and promotion of systemic inflammation [2]. 

Previous studies showed an association of OSA with an increased risk of cardiovascular and cerebrovascular diseases, stroke, coronary artery disease, diabetes, hypercholesterolemia and hypertension [5,6,7,8]. Furthermore, OSA has been shown to be a major risk factor concerning optic nerve disorders, such as glaucoma, non-arteritic anterior ischemic optic neuropathy, central serous chorioretinopathy, papilledema and retinal vein occlusion [9,10,11]. The term “glaucoma” describes a collection of progressive, chronic optic neuropathies resulting from the degeneration of retinal ganglion cells and leading to visual impairment. On funduscopic examination, a cupping of the optic disc is found. Glaucoma can be divided into different clinical subgroups: primary open angle glaucoma (POAG), angle-closure glaucoma (ACG) and normal tension glaucoma (NTG) [12,13]. The pathophysiology of glaucomatous changes is not completely known. The most frequent subgroup is POAG, which is associated with a high IOP. Hence, IOP is the main clinical endpoint accessible to therapeutic intervention [13].

The relationship between OSA and glaucoma is not yet fully understood. Several studies indicate that a change in the intraocular pressure during apnea phases leads to glaucomatous changes in OSA patients. In these studies, contact lens sensors were used for continuous IOP-monitoring. Shinmei et al. found a statistically significant decline in the IOP during apneas compared to non-apneic phases in four out of seven patients with suspected OSA. In association with apneic phases, the CLS-based IOP values dropped by 23.1 ± 16.4 mV eq on average [14]. Carnero et al. examined 20 participants with suspected OSA. The patient cohort was split into severe and mild/moderate OSA. In contrast to Shinmei et al., patients with severe OSA showed a longer IOP elevation compared to patients with a mild/moderate disease (*p* = 0.032/*p* = 0.028) [15]. Fang et al. investigated the differences in the IOP values during and after varying body positions in OSA and non-OSA patients. The IOP was measured ten minutes after sitting, directly and 30 min after changing to supine position and again after resuming sitting position. OSA patients showed an increase in the IOP when changing from sitting to supine position (right eye *p* = 0.033, left eye *p* = 0.044) and a further increase after 30 min in supine position (right eye *p* = 0.001, left eye *p* = 0.246). A higher IOP in supine position was associated with a higher IOP in sitting position post-supine and the authors concluded that the IOP rises more in OSA patients in prolonged supine position [16]. 

This review concentrates on the causality and mutual influence of glaucoma and OSA, as well as the mutual influences of the respective therapies. The most recently published systematic review discussing the connection between OSA and glaucoma, that by Wu et al. [17], included papers up to 2014.

## 2. Materials and Methods

This review was conducted in accordance with the Preferred Reporting Items for Systematic Reviews and Meta-Analyses (PRISMA) guidelines.

Literature Search

**Figure 1:**
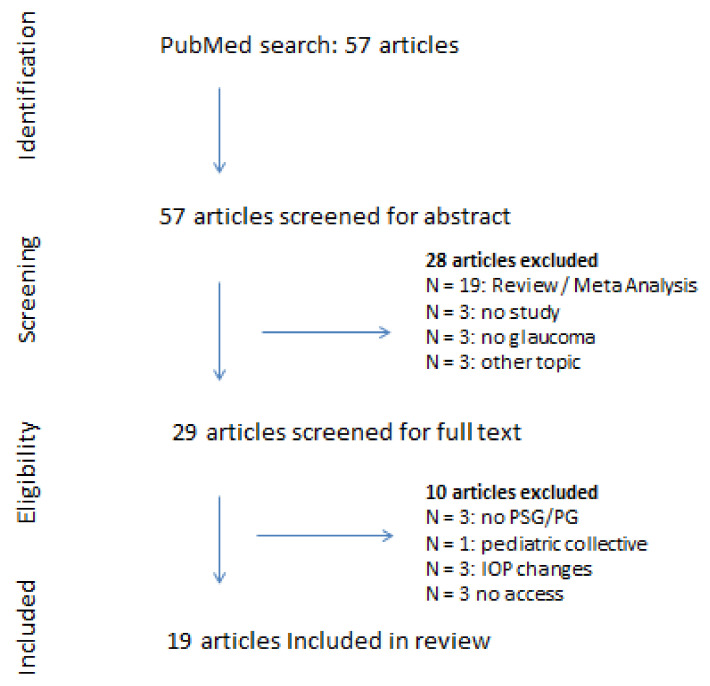
Literature selection process.

PubMed was searched using the Medline terms: [(obstructive sleep apnea) OR (OSA) AND (glaucoma) AND (“2015/01/01” [PDat]: “2020/31/12” [PDat])]. A title/abstract screening was followed by a full text screening. Only articles written in English or German were screened. Eligible studies were original articles such as case–control studies, cohort studies, cross-sectional studies and randomized controlled trials. Animal studies were not included. Only studies defining OSA using the AHI via polysomnography or home sleep-apnea testing (HSAT/polygraphy) were included. Studies were included that used at least one of the following ophthalmic examination methods regarding glaucoma: optical coherence tomography imaging (OCT), measurement of IOP, sonography and perimetry for measurement of the visual field (VF). Literature published from 2015 until the end of 2020 was reviewed to exclude already published data from the last systematic review by Wu et al. [17] and therefore avoid publication bias. Figure 1 shows the literature selection process.

## 3. Results

### 3.1. Cross-Sectional Studies on the Occurrence of Glaucoma in Sleep Clinic Cohorts

A complete list of the studies is provided in Table 1.

In a cross-sectional case series study including 83 participants, Bagabas et al. [18] compared the prevalence of glaucoma in OSA patients with that of the standard population. The measured IOP of both eyes was higher in individuals with OSA than in the control group (left eye: 14.8 ± 3.3 vs. 13.1 ± 3.3, *p* = 0.023, right eye: 14.5 ± 3.4 vs. 13 ± 3.7, *p* = 0.052). The mean cup/disk ratio was similar in both groups (left eye: 0.24 ± 0.13 vs. 0.25 ± 0.22, *p* = 0.760, right eye: 0.25 ± 0.15 vs. 0.24 ± 0.20, *p* = 0.892). Only one patient had an IOP > 21 mmHg (OSA group). Sixteen percent of patients with OSA presented with comorbid glaucoma and eight percent of the participants without OSA presented with glaucoma (*p* = 0.267), which can only be seen as a trend. 

In the retrospective study by Chuang et al. [19], the prevalence of normal tension glaucoma in 53 OSA patients was evaluated. RNFL thickness decreased in moderate/severe OSA compared to mild OSA (88.72 ± 13.58 vs. 96.64 ± 10.64, *p* = 0.05) without statistical significance. The GCC thickness decreased significantly when comparing both groups (88.13 ± 11.89 vs. 96.28 ± 6.34, *p* = 0.003). There was no difference in IOP for both groups (15.32 ± 2.50 vs. 15.67 ± 3.14, *p* = 0.68). The AHI was significantly higher in patients with NTG than in patients without NTG (45.39 ± 18.34 vs. 31.55 ± 25.15, *p* < 0.001). In conclusion, the study showed a high AHI to be a risk factor for NTG in an OSA population. The authors also concluded that OCTA is a good monitor for ophthalmic microcirculation in OSA because the vascular changes were highly correlated with the visual field defects.

In a study from 2020, Lee et al. [20] investigated a middle-aged/older patient collective. There was no significant difference in RNFL thickness in patients with and without OSA (*p* > 0.13), but patients with severe OSA had significantly thinner RNFL superotemporally compared to patients without OSA (*p* < 0.001) and with mild OSA (*p* = 0.001). There was no significant difference in superotemporal RNFL thickness in no, mild and moderate OSA groups or between the moderate and severe OSA groups (*p* = 0.021). The superotemporal RNFL became thinner by about 0.9 μm with every increase in the AHI of five events per hour (*p* = 0.004) and by 34 μm with every increase of one percent in T90% (*p* = 0.005).

Morsy et al. [21] found a higher risk to develop glaucoma in OSA patients. Twenty-four of the eighty OSA patients presented with glaucoma (30%), and there was a seven times greater risk of developing vision-threatening disorders, such as glaucoma, senile cataract and retinal ischemia. AHI, basal oxygen level, lowest oxygen saturation index, desaturation index, total severity index and arousal index were significantly associated with the occurrence of glaucoma (*p* = 0.001). OSA patients with NTG showed a significantly thinner RNFL and a higher cup/disk ratio (both *p* = 0.001).

In a study conducted by Moyal et al. [22], OCT scans did not detect significant modifications in OSA patients. There were no statistically significant differences between all groups (no, mild, moderate, severe OSA) concerning IOP and CCT (*p* = 0.55, *p* = 0.83). There were also no statistically significant differences in RNFL thinning and cup/disk ratio (*p* > 0.156, *p* = 0.333). Patients with severe OSA had an altered mean defect in VF (*p* = 0.014). 

Pedrotti et al. [23] found a higher prevalence of glaucoma in OSA patients (11.1%) in a cohort of 296 patients. Severe OSA was significantly associated with occurrence of glaucoma (*p* = 0.037). Therefore, patients with previously diagnosed or suspected OSA should be thoroughly assessed by an ophthalmologist. 

In 2018, Swaminathan et al. [24] did not find a higher risk for glaucoma progression in OSA patients. Of the 25 enrolled patients with OSA, 11 were classified as progressors and 14 as non-progressors. Progressors and non-progressors had similar AHI values (31.3 ± 18.6 vs. 26.4 ± 24.0, *p* = 0.58) and the OSA severity was not significantly associated with glaucoma progression (*p* = 0.41). No statistically significant correlations were identified between AHI and MD, pattern standard deviation (PSD) or visual field index (VFI, *p* = 0.190, *p* = 0.312, *p* = 0.228). Compared to non-progressors, the odds ratio of being a progressor with an increase in AHI of five events per hour was 1.06 (95% CI: 0.09–1.3).

Furthermore, no higher prevalence of glaucoma in OSA patients was found by Wozniak et al. [25]. OSA was diagnosed in 58% of POAG patients and in 54% of control patients (*p* = 0.44). The AHI was not a significant predictor of the glaucoma severity stages measured by visual field loss (*p* = 0.40). There was also no significant association between AHI and RNFL thinning (*p* = 0.97 adjusted) and between OSA and non-OSA groups (*p* > 0.2). The authors therefore did not recommend a general screening for OSA in POAG patients.

### 3.2. Cross-Sectional Studies of the Occurrence of OSA in Patients with Glaucoma

Fan et al. [26] investigated the correlation between severity of OSA and glaucoma progression. Fifty-three patients with POAG, NTG or suspected glaucoma were included. A high severity of OSA was non-significantly correlated with a higher percentage of RNFL thinning progression (*p* = 0.096). When combining groups into no/mild OSA and moderate/severe OSA, the difference in RNFL thinning progression between both groups was statistically significant (64.7% vs 26.7%, *p* = 0.042). A higher severity of OSA was non-significantly correlated with a higher percentage of progression in the visual field (VF, *p* = 0.219). The patients in the moderate/severe OSA group showed a higher percentage of progression in the VF compared to the no/mild OSA group, without statistical significance (17.6% vs 0.0%, *p* = 0.229). After adjustment for different cofactors (age, sex, diabetes mellitus, hypertension, hyperlipidemia and BMI) the multivariate Cox regression analysis showed that severe OSA had an 8.448-fold higher risk of RNFL thickness progression than no or mild OSA did (95% CI 1.464–48.752, *p* = 0.017). According to the authors, undiagnosed severe OSA should be taken into consideration in patients with glaucoma progression despite adequate ophthalmological treatment (Table 2). 

### 3.3. Cross-Sectional Studies and Chart Reviews to Evaluate Associations between OSA and Glaucoma in the General Population

In 2019, Lee et al. [27] found that young OSA patients showed a thinner RNFL at the inferotemporal and superotemporal segments compared with those without OSA (*p* = 0.026 and *p* = 0.008). Patients with mild OSA showed significantly lower RNFL values at the inferotemporal and superotemporal segments (*p* = 0.033, *p* = 0.028) compared with those without OSA. The moderate or severe OSA group did not differ significantly from the mild/no OSA group. There was also no significant difference found between no/mild OSA and moderate/severe OSA. An increase in the AHI by five events per hour was significantly associated with thinning of the RNFL by 1.5 µm superotemporally (*p* = 0.007).

Friedlander et al. [28] conducted a retrospective case review including 225 OSA patients, comparing them to 312,494 patients without OSA in terms of POAG/NTG prevalence. A total of 47 of the 225 participants were diagnosed with POAG (20.9 %) and 7975 patients without OSA presented with POAG (2.5 %, *p* < 0.00001). No statistically significant differences in the prevalence in the different OSA groups defined by AHI were found using logistic regression models. The authors concluded that patients with a recently diagnosed sleep breathing disorder who are to undergo surgery should be seen by an ophthalmologist at the perioperative stage to avoid potential vision loss (Table 3).

### 3.4. Prospective Studies of Glaucoma Incidence or Changes in Intraocular Pressure in Patients with OSA

Abdullayev et al. [29] conducted a prospective study including 59 patients with OSA (*n* = 28 with CPAP) and 19 controls to evaluate the prevalence of glaucoma in OSA patients using and not using CPAP compared to controls. Based on the AHI, all patients with OSA were divided into three groups: 19 patients with mild OSA (32.2 %), 16 patients with moderate OSA (27.1 %) and 24 patients with severe OSA (40.67 %). Average retinal nerve fiber layer (RNFL) thickness values in the right and left eyes were 91.0 ± 10.3 µm and 89.7 ± 10.3 µm (mild OSA = group one), 93.2 ± 7.01 µm and 89.6 ± 8.5 µm (moderate OSA = group two), 95.5 ± 10.4 and 93.1 ± 8.7 (severe OSA = group three) and 95.2 ± 9.8 and 95 ± 8.6 (control group). No statistically significant difference was found (*p* > 0.05) between the groups. Only in group two was a statistically significant correlation between AHI and average RNFL thickness of the left eye found (*p* = 0.010). Furthermore, the ganglion cell complex (GCC) thickness was examined. In comparison to the control group, group one showed significantly lower values in the inferior and inferonasal sectors of both eyes (*p* = 0.029, *p* = 0.022, *p* = 0.037 and *p* = 0.019). The average GCC and minimum GCC for the left eyes were significant lower in all OSA groups compared to the control group (*p* = 0.013, *p* = 0.010, *p* = 0.019, *p* = 0.004). The authors concluded that a periodic evaluation of RNFL and GCC thickness could have diagnostic value in early identification of glaucoma in OSA patients.

Bahr et al. [30] carried out a prospective study to assess the risk for glaucoma and ocular hypertension in patients with OSA. A total of 101 patients with suspected glaucoma were screened for OSA. The diagnosis of glaucoma was confirmed in 87 patients, the control group comprised 14 participants without glaucoma. OSA was significantly more prevalent in patients with a high IOP: the median AHI in POAG was 22.7 events per hour and 21.9 events per hour in the OH group. With regard to the AHI, highly significant differences were found between the four groups (Chi2 = 22, df = 3, *p* < 0.0001), with lower values in the LTG group compared to the POAG group (Hodges–Lehmann = −13.8, 95% CI (−18.6–−8.8; *p* < 0.0001) and the control group (Hodges–Lehmann = 12.1; 95% CI −19.9–−2.4; *p* < 0.02). Of the 53 POAG patients, 48 presented with OSA (11 = mild, 23 = moderate, 14 = severe). In conclusion, the study supported the hypothesis that OSA leads to a rising IOP and, therefore, a higher risk risk of POAG and OH.

A prospective, randomized study by Findik et al. [31] included 60 patients examined via spectral-domain optical coherence tomography and investigated whether an association between OSA and glaucomatous optic neuropathy exists. Sixteen patients formed the control group and forty-four were diagnosed with OSA (14 mild, 15 moderate and 15 severe OSA). Furthermore, the ophthalmic, retinal and posterior ciliary artery pulsatile index (PI) and resistive index (RI) were measured by coloured Doppler sonography. In the severe OSA group, RNFL thickness values decreased significantly in mean values and in superior and inferior quadrants compared to the other groups (*p* = 0.026, *p* = 0.046 and *p* = 0.024). No significant differences were observed between all four groups in the RNFL thickness values of the nasal and temporal quadrants (*p* > 0.05). There was a negative correlation between AHI and mean values and superior and inferior RNFL thickness values (r = −0.313, *p* = 0.015; r = −0.3, *p* = 0.02; r = −0.278, *p* = 0.032), while there was a positive correlation with IOP values (*r* = 0.472, *p* < 0.01). The PI and RI in the posterior ciliary artery were statistically significantly higher in the severe OSA group (*p* < 0.05). Due to its smaller diameter, the PCA is more susceptible to stenosis, and this can lead to optic nerve ischemia and an ensuing decrease in RNFL thickness.

A prospective study conducted by Gross et al. [32] could not find a higher prevalence of glaucoma in OSA patients. Of the 100 included patients, 78 were diagnosed with OSA (14 mild, 23 moderate, 41 severe). The prevalence of glaucoma in their OSA cohort was 2%. There was no statistically significant correlation between OSA severity and IOD, VF and cup/disk ratio. Nevertheless, the authors plead for PSG in glaucoma patients presenting with OSA symptoms and also an ophthalmological examination in OSA patients.

Teberik et al. [33] conducted a prospective case–control study including 103 patients with OSA. The mean IOP values of patients with OSA were non-significantly lower than those of the control group. It was also found that the differences between the mean values of the RNFL thickness in all quadrants were similar in both OSA and control groups (*p* = 0.274). When splitting the OSA group in mild, moderate and severe OSA, no statistically significant differences in RNFL and IOP were found. 

In summary, three of the five reviewed studies showed an association between OSA and glaucomatous changes, while the other two studies failed to show a correlation between OSA and glaucoma. Of the abovementioned studies, two showed alterations in GCC and in RNFL thinning in OSA patients. One study supported the hypothesis that OSA leads to a rising IOP and, hence, a higher risk of POAG and OH (Table 4).

### 3.5. Effect of OSA Treatment on Intraocular Pressure

The gold standard therapy for OSA treatment is CPAP. Several studies investigated the influence of CPAP on IOP. Another treatment option for OSA is upper airway surgery to prevent upper airway collapse. The following studies compared IOP values of OSA patients treated with one of those therapy options.

Abdullayev et al. [29] compared ophthalmological parameters of CPAP users, OSA patients without CPAP and controls. There were no statistically significant differences in IOP, CCT and cup/disk ratio in the three groups. The MD values of left eyes in the non-CPAP group were significantly higher than those of the control group (*p* = 0.054). The RNFLs in the nasal quadrants of left eyes were significantly thinner in the non-CPAP group than in the control group (*p* = 0.047). The average RNFL thickness values did not differ significantly (CPAP 93.38 ± 8.54, non-CPAP 94.10 ± 10.09, no OSA 95.21 ± 9.80, *p* = 0.817).

In 2019, Fan et al. [26] investigated the influence of CPAP and upper airway surgery on ophthalmological parameters. Of 27 OSA patients, 7 were treated with CPAP and 2 patients had surgery. A progression of RNFL values was seen in 66.7% of patients with treatment and in 44.4% of patients without treatment (*p* = 0.420). Furthermore, no statistically significant differences in the progression of MD and VF in OSA patients treated and not treated were found. The authors are conducting another study to investigate the neuroprotective effects of OSA treatment in glaucoma patients.

Hirunpatravong et al. [34] included 6 patients in a prospective study to evaluate the long-term effects of CPAP on IOP. The average AHI was 37.42 ± 15.62/h before treatment and improved to 2.53 ± 1.55/h after 12 months of CPAP. Furthermore, twelve months after initiation of CPAP therapy, the mean IOP significantly increased compared to that at the baseline (19.08 ± 3.47 vs. 17.83 ± 2.89 mmHg, *p* = 0.006). There were no statistically significant differences in MD and VFI before and after 12 of months of CPAP therapy. In conclusion, a risk of glaucoma progression in CPAP users could not be verified or falsified and the authors recommend IOP monitoring and regular IOP screening in CPAP users.

In a prospective, single-blinded study from 2019, Lin et al. [35] examined the effect of upper airway surgery on ophthalmological values in 108 patients. In the mild/moderate group, VFI, IOP and RFNL thickness values did not change significantly before and after treatment. MD values in VF decreased significantly after treatment (−2.17 ± 1.54 vs. −1.24 ± 1.19, *p* = 0.0001). In the severe OSA group, MD and VFI changed significantly after upper airway surgery (−2.15 ± 1.75 vs. −1.17 ± 1.08, *p* < 0.0001, 97.9 ± 3.1 vs. 99.0 ± 1.3, *p* = 0.0009). No statistically significant differences were observed in IOP and RNFL thickness values.

Swaminathan et al. [24] also investigated the influence of CPAP on their patient collective. During the course of follow-up (mean 1.3 years after initiation of CPAP), the mean IOP after initiation of CPAP therapy was similar in glaucoma progressors (*n* = 6, IOP 14.2 ± 3.3 mmHg) and non-progressors (*n* = 7, IOP 13.9 ± 2.7 mmHg, *p* = 0.85). The authors stated that a final statement on the impact of CPAP on glaucoma progression cannot be made due to the short use time of CPAP.

A study from 2015 compared ophthalmological parameters of patients without OSA to those of OSA patients using a CPAP device and non-users [36]. The IOP was significantly higher in patients not using CPAP compared to those using CPAP, and the IOP in the CPAP was group was similar to the OSA-free group (mean IOP 15.1 ± 3.5, median 14.0, 16.7 ± 3.1, 17.0, 14.1 ± 2.4, 14.0, *p* = 0.000). The same results were seen in terms of cup/disk ratio (mean ratio 0.4 ± 0.1, median 0.3, 0.4 ± 0.2, 0.5, 0.3 ± 0.1, 0.3, *p* = 0.000). Glaucoma prevalence was 5.2% in the CPAP group, 12.5% in the non-CPAP group and 0% in the OSA-free group. Ulusoy et al. concluded that CPAP has positive and healing effects on glaucoma.

In conclusion, one study out of three surveying specific ophthalmological parameters showed an influence of OSA therapy on RNFL thinning and vision. One study showed an increase in IOP, while two other studies showed no increase under CPAP (in progressors and non-progressors) (Table 5). 

## 4. Discussion

This narrative review aimed to summarize the current findings on the association of glaucoma and obstructive sleep apnea. Studies investigating the association and prevalence of both entities, as well as studies that focused on the evolution of glaucoma parameters under OSA treatment, were analyzed.

Overall, a total of 19 studies with 316,178 participants were included in this review [18,19,20,21,22,23,24,25,26,27,28,29,30,31,32,33,34,35,36]. The diagnosis of OSA was based on the AHI measured using polysomnography or home sleep-apnea testing. The main features of all studies are presented in Table 1, Table 2, Table 3, Table 4 and Table 5.

The results of the included studies are often contradictory. Therefore, an association between OSA and glaucoma remains controversial. Eight cross-sectional studies investigating the occurrence of glaucoma in sleep clinic cohorts were included [18,19,20,21,22,23,24,25]. There appears to be a higher risk of glaucoma prevalence among OSA patients. Five of the eight reviewed studies showed an association of OSA and glaucomatous changes. Four of these studies showed a correlation between OSA severity (AHI) and the occurrence of glaucoma and one study correlated the lowest oxygen saturation to the prevalence of glaucoma. Of the abovementioned studies, one showed alterations in GCC and three in RNFL thinning in OSA patients. Three of the eight included studies did not show a correlation between OSA and glaucoma. Due to the character of the included studies, the association found can only be seen as a trend. 

One cross-sectional study was included investigating the occurrence of OSA in a glaucoma cohort. They assessed a correlation between severity of OSA and glaucoma progression [26]. After adjustment for different cofactors, the analysis showed that severe OSA had an 8.448-fold higher risk of RNFL thickness progression than no or mild OSA did. This can also only be seen as a trend, but it might be a useful tool to test patients with glaucoma progression despite adequate ophthalmological treatment and with OSA-specific symptoms via PG or PSG.

Two studies investigated the associations between OSA and glaucoma in a general population cohort (one cross-sectional study and one retrospective chart review) [27,28]. The first study showed that an increase in the AHI by five events per hour was significantly associated with a thinning of the RNFL by 1.5 µm superotemporally and the second failed to show a significant association between both entities. 

Since none of the abovementioned studies were prospective, randomized, clinical trials, conclusions about the causality between glaucoma and OSA cannot be drawn.

In this review, five prospective studies of incident glaucoma or changes in intraocular pressure in patients with untreated OSA were included. Three of them showed a statistically significant relationship between glaucoma and OSA. Only one of the studies was randomized. In particular, the thinning of the RNFL seen via OCT correlated with the occurrence of OSA. Two of the abovementioned prospective studies showed a statistically significant association of thinning of the RNFL, or specific quadrants, with OSA. Glaucomatous optic neuropathy is associated with thinning of the RNFL and this is one of the first visible signs on OCT scans, followed by progression of optic disc excavation, leading to changes in the visual field [37,38]. Only two of the above discussed studies showed statistically significant visual field defects in patients with OSA. Therefore, according to the current evidence, an ophthalmological examination using OCT appears to be the best option for early detection of glaucomatous changes in OSA patients. 

The only therapeutic approach to treating glaucoma is lowering the IOP. In a preceding prospective study, CPAP therapy was associated with rising IOP [39]. Six recent studies were included that investigated the effect of OSA therapy on different ophthalmological outcomes. Abdullayev et al. [29] and Swaminathan et al. [24] found no statistically significant differences in IOP before and after initiation of CPAP therapy. 

Hirunpatravong et al. [34] conducted a prospective study including six POAG patients with newly diagnosed OSA. After initiation of CPAP therapy, the patients developed significant rising IOP in comparison to the baseline IOP, but did not show progression of glaucoma. In contrast, Ulusoy et al. [36] included 38 patients using CPAP devices, 32 OSA patients not using CPAP devices and 36 healthy controls. The IOP was significantly lower in the CPAP group than in the non-CPAP group but similar to the control group. No effect on IOP was seen in the patient cohort studied by Lin et al. [35]. They included 108 patients who underwent upper airway surgery as OSA treatment. However, visual sensitivities in SAP, ML thickness in OCT and oxygenation status in PSG significantly improved 6 months after upper airway surgery in severe OSA patients. Four of those studies had a relatively small patient collective (6–28 patients) and, therefore, the evidence is not conclusive. The two larger studies showed promising ophthalmological data after initiation of CPAP therapy and also after upper airway surgery in severe OSA. 

Another interesting link between OSA and glaucoma is hypoxia with upregulation of HIF-1α. The occurrence of intermittent hypoxia, as in OSA patients, leads to upregulation of HIF-1α protein (which activates the transcription of genes coding for erythropoietin and endothelial vascular growth factor) [40]. This upregulation is dependent on the severity of hypoxemia [41]. HIF-1α expression was also found in the retina and optic nerve of glaucomatous eyes [42,43]. It is possible that HIF-1α is not only helpful in classifying and monitoring OSA but can also apply as a possible therapeutic approach to treat glaucoma. 

Hitherto, the severity of OSA has been classified using the AHI (and not serum proteins, such as HIF-1α). The AHI, on the other hand, as a continuous variable, does not always correlate with glaucoma-specific parameters. Since using the AHI as the only OSA-defining parameter is controversial among sleep medicine experts, it would not be surprising if it was not able to adequately depict a possible interaction with glaucoma-specific parameters. Most of the included studies examined a correlation between AHI and glaucoma. Some studies also investigated a possible correlation between median or minimum nocturnal oxygen saturation, oxygen desaturation index (ODI) or sleep time with oxygen saturation level below 90% (T90%). 

Bahr et al. [30] found a higher ODI in POAG patients than in LTG patients or healthy controls. Chuang et al. [19] detected a higher ODI in NTG patients compared with controls, and Morsy et al. [21] showed the lowest oxygen desaturation index to be a significant predictor of vision-threatening disorders. Lee et al. [20] found that the superotemporal RNFL was inversely associated with T90% and that it was thinner by 34 μm with every increase of one percent in T90%. In another study by Lee et al. [27], a correlation between a lower oxygen saturation and RNFL thickness could not be found. In their cohort comprising only young adults, the range of the SpO2 nadir was narrow (87–93%), as expected in young adults, and, therefore, the authors concluded that a meaningful analysis was limited. This depicted cohort will be followed up through the decades to document any further optic disc changes in relationship to OSA parameters. 

Our findings suggest an association of OSA and glaucoma, although a causal relationship of both entities cannot be ascertained due to the lack of prospective, randomized studies. This hypothesis is congruent with a meta-analysis from the year 2015 by Wu et al. [17], who included 12 studies with 36,909 subjects on the association between OSA and glaucoma risk (OR = 1.65; 95% CI). Patients with severe OSA had significantly higher risk of developing glaucoma (OR = 5.49; 95% CI) than patients with mild or moderate OSA and OSA patients showed an increased risk of developing POAG (OR = 1.87; 95% CI) but not NTG (OR = 3.57; 95% CI).

The present study is not a meta-analysis, so the direct comparability of the included studies is not given, which is the main limitation. We included studies from January 2015 until March 2021 and, thus, the studies analyzed by Wu et al. [17] did not overlap with ours. 

## 5. Conclusions

In conclusion, our findings suggest an association between OSA and glaucoma and especially OSA and RNFL thinning as an early detector of glaucoma. The findings can also be seen as a trend and direct causality is still not proven. Since there is controversy regarding the increase of IOP from the use of CPAP in the included studies, we suggest that further studies are needed to investigate this relationship. On the basis of the recent findings, CPAP and upper airway surgery can also be recommended to OSA patients with comorbid glaucoma. Due to the lack of randomized controlled trials, the effect of CPAP on glaucomatous changes, especially the IOP, can only be insufficiently predicted. The AHI, in particular, was not always correlated with glaucoma. Therefore, we recommend that other polysomnographic measurements should be taken into account when screening for OSA in glaucoma patients.

## Figures and Tables

**Table 1:** Cross-sectional studies of the occurrence of glaucoma in sleep clinic cohorts.

Reference	Population	Eligibility	Intervention	Control Group	Study Design	Follow-Up	Findings	Correlation Found
**Bagabas et al. [18]**	*n* = 83 *n* = 44 OSA	PSG, VF, OCT, IOP	-	*n* = 39 no OSA	Cross- sectional case series study	-	Glaucoma prevalence was higher among individuals with OSA (16%) than among non-OSA individuals (8%, *p* = 0.267). A consistent trend towards more glaucomatous changes was observed in OSA subjects.	Yes
**Chuang et al. [19]**	*n* = 53 OSAS	PSG, OCT(A), VF	-	-	Retrospective cross-sectional study	-	There was significantly higher AHI in the NTG group (*n* = 27) than in the control group (*n* = 26; *p* < 0.001). Superficial and deep-layer peripapillary and macular area VD significantly decreased in the NTG group.	Yes
**Lee et al. [20]**	*n* = 865, *n*= 411 OSA	PG, OCT	-	*n* = 454 no OSA	Cross-sectional study plus follow-up	6 years	Participants with severe OSA had thinner RNFL superotemporally than those without or with mild OSA (*p* < 0.001 and 0.001). Superotemporal RNFL was inversely associated with AHI (*p* = 0.004) and T90% (*p* = 0.005).	Yes
**Morsy et al. [21]**	*n* = 100, *n* = 80 OSA	PSG, IOP, VF, OCT	-	*n* = 20 no OSA	Cross-sectional case control study	-	Glaucoma was diagnosed in 24 out of 80(30.0 %) patients. There was a higher risk to develop glaucoma among OSA and the lowest oxygen saturation was significantly associated with vision-threatening disorders (NTG, senile cataract and retinal ischemia, *p* = 0.001).	Yes
**Moyal et al. [22]**	N = 53 OSA	PSG, OCT(A), VF, IOP	-	*n* = 28 no OSA	Retrospective observational study	-	OCTA did not detect reduced ONH, RPC, or macular blood vessel density in eyes with OSA. RNFL thickness, cup/disc ratio, rim area, and GCC were not significantly modified.	No
**Pedrotti et al. [23]**	*n* = 296 OSA	PSG, IOP, OCT, VF	-	-	Cross-sectional cohort study	-	Severe OSA was significantly associated with glaucoma (OR, 95% CI 1.05 to 5.93, *p* = 0.037). A total of 11.1% (*n* = 33) of OSA patients had glaucoma.	Yes
**Swaminathan et al. [24]**	*n* = 25 OSA + glaucoma, *n* = 13 CPAP	PSG, VF, IOP	CPAP	-	Retrospective cross-sectional study	>2 years, (PSG within 12 months of final VF)	Progressors and non-progressors had non-significantly different IOP (13.1 ± 2.8 vs. 14.9 ± 2.5 mm Hg), mean ocular perfusion pressure (49.7 ± 5.5 vs. 48.8 ± 9.0 mm Hg) and AHI (31.3 ± 18.6 vs. 26.4 ± 24.0). AHI was not correlated with slopes of VF mean deviation (*p* = 0.190) or pattern standard deviation (*p* = 0.312), and no substantial increase in risk of progression was found with increases in AHI (independently of CPAP)	No
**Wozniak et al. [25]**	*n* = 235 POAG	PG, OCT, VF, CCT	-	*n* = 160 no POAG	Case control study	-	There was no significant difference in OSA prevalence between the matched groups (*p* = 0.91 for AHI ≥ 5 and *p* = 0.66 for AHI ≥ 15). The AHI was not associated with the severity of visual field defect or RNFL thinning after adjustment for confounders.	No

AHI = Apnea–Hypopnea Index; CCT = central corneal thickness; CPAP = continuous positive airway pressure; GCC = ganglion cell complex; IOP = intraocular pressure; LTG = low tension glaucoma; MD = mean deviation; NTG = normal tension glaucoma; OCT (A) = optical coherence tomography angiography; OH = ocular hypertension; ONH = optic nerve head; OSA = obstructive sleep apnea; PG = polygraphy; PSG = polysomnography; RNFL = retinal nerve fiber layer; RPC = radial peripapillary capillary; VF = visual field.

**Table 2:** Cross-sectional studies on the occurrence of OSA in patients with glaucoma.

Reference	Population	Eligibility	Intervention	Control Group	Study Design	Follow-Up	Findings	Correlation Found
**Fan et al. [26]**	*n* = 32 POAG/NTG/suspect	PSG, IOP, VF, OCT	*n* = 7 CPAP, N = 2 upper airway surgery	*n* = 5 no OSA, *n* = 7 glaucoma suspect	Comparative cohort study	>3 years	A more severe OSA was associated with a higher percentage of progression of glaucoma (*p* = 0.017).	Yes

CPAP = continuous positive airway pressure; IOP = intraocular pressure; OCT = optical coherence tomography; OSA = obstructive sleep apnea; PSG = polysomnography; VF = visual field.

**Table 3:** Cross-sectional studies and chart reviews to evaluate associations between OSA and glaucoma in the general population.

Reference	Population	Eligibility	Intervention	Control Group	Study Design	Follow-Up	Findings	Correlation Found
**Lee et al. [27]**	*n* = 848, *n* = 178 OSA	OCT and after 2 years PSG	-	*n* = 670 no OSA	Cross-sectional cohort study	Planned	Participants with OSA showed thinner peripapillary RNFL inferotemporally (*p* = 0.026) and superotemporally (*p* = 0.008) compared to those without. A higher AHI was associated with thinner RNFL superotemporally (*p* = 0.007). There were no significant differences in optic disc measures between groups of OSA severity.	Yes
**Friedlander et al. [28]**	*n* = 225 OSA	PSG/ alio loco, VF, IOP	-	*n* = 312,494 no OSA	Retrospective case review	-	The POAG prevalence rate among the OSA group (20.9 %) was significantly higher than among the medical center’s general population (2.5%, *p* < 0.00001). Severity of OSA (AHI) failed to demonstrate a significant correlation to any POAG subtype (*p* > 0.05).	

AHI = Apnea–Hypopnea Index; IOP = intraocular pressure; OSA = obstructive sleep apnea; PSG = polysomnography; POAG = primary open angle glaucoma; VF = visual field.

**Table 4:** Prospective studies of incident glaucoma or changes in intraocular pressure in patients with OSA.

Reference	Population	Eligibility	Intervention	Control Group	Study Design	Follow-Up	Findings	Correlation Found
**Abdullayev et al. [29]**	*n* = 59 OSA, *n* = 28 with CPAP	PSG, OCT, IOP, VF	-	*n* = 19 without OSA	Prospective study	-	Average GCC thickness was significantly lower in mild OSA than in controls (left eye, *p* = 0.013). The GCC was significantly thinner in the inferior and inferonasal sectors of both eyes in OSA compared to controls (*p* = 0.029, *p* = 0.022, *p* = 0.037, and *p* = 0.019). Minimum GCC thickness in the left eyes of all OSA groups was significantly lower than in the control group (*p* < 0.05).	Yes
**Bahr et al. [30]**	*n* = 101 glaucoma/OH	PG, IOP, VF	-	*n* = 14 no glaucoma/OH	Prospective study	-	There was a strong correlation between POAG and OH clinical glaucoma phenotypes and the AHI. LTG patients had a significantly lower rate of OSA compared to other glaucoma types and controls.	Yes
**Findik et al. [31]**	*n* = 60 *n* = 44 OSA	PSG, OCT, sonography, VF	-	*n* = 16 no OSA	Prospective randomized study	-	Superior and inferior RNFL thickness values were significantly lower than those in the control group (*p* < 0.046). Glaucoma prevalence of OSA patients in this study was 6.8% (only in the severe OSA group).	Yes
**Gross et al. [32]**	*n* = 100, (*n* = 22 CPAP)	PSG, IOP, VF	-	-	Prospective study	-	There was no higher prevalence of glaucoma in OSA. No statistically significant correlation (ANOVA) was found between RDI, IOP, MD and cup-disc ratio.	No
**Teberik et al. [33]**	*n* = 103 OSA	PSG, IOP, sonography (CCT), OCT	-	*n* = 37 without OSA	Prospective case–control study	-	The mean values of the RNFL thickness in all quadrants were not different in the OSA and control group (*p* = 0.274). The IOP and CCT measurement averages in the OSA were lower than the control group (no statistical significance).	No

AHI = Apnea–Hypopnea Index; CCT = central corneal thickness; CPAP = continuous positive airway pressure; GCC = ganglion cell complex; IOP = intraocular pressure; LTG = low tension glaucoma; OCT = optical coherence tomography; OH = ocular hypertension; OSA = obstructive sleep apnea; PG = polygraphy; PSG = polysomnography; POAG = primary open angle glaucoma; RNFL = retinal nerve fiber layer; VF = visual field.

**Table 5:** Effect of treatment of OSA on intraocular pressure.

Reference	Population	Eligibility	Intervention	Control Group	Study Design	Follow-Up	Findings	Influence
**Abdullayev et al. [29]**	*n* = 59 OSA, *n* = 28 with CPAP	PSG, OCT, IOP, VF	CPAP	*n* = 19 without OSA	Prospective study	-	There was no statistically significant difference in CCT and RNFL values between OSA with and without CPAP and the control group. The mean deviation value in left eyes in non-CPAP was significantly higher than that of the control group (*p* = 0.054). Mean PSD values in the right eyes of CPAP and non-CPAP were significantly higher than those of the control group (*p* = 0.016 and *p* = 0.014).	No
**Fan et al. [26]**	*n* = 32 POAG/NTG/suspect	PSG, IOP, VF, OCT	*n* = 7 CPAP, N = 2 upper airway surgery	*n* = 5 no OSA, N = 7 glaucoma suspect	Comparative cohort study	>3 years	There were no statistically significant differences for progression, RNFL thickness, MD and VFI in patients treated with CPAP/surgery in comparison to the no-treatment group.	No
**Hirunpatravong et al. [34]**	*n* = 6 POAG and OSA	PSG, VF, IOP, sonography	CPAP	-	Prospective study	IOP every 3 months, VF at baseline and 12 months	POAG and OSA patients demonstrated significant IOP increases after CPAP therapy (*p* = 0.006) but did not show progression of glaucomatous damage. MD, PSD, and VFI were not significantly different after CPAP therapy.	Yes but no progression
**Lin et al. [35]**	*n* = 108 OSA	PSG, OCT	Upper airway surgery	-	Prospective single-blind study	Baseline, 6 months after surgery	The visual sensitivities for SAP, ML thickness in OCT, and the oxygenation status in PSG, significantly improved 6 months after upper airway surgery in patients with severe OSA.	Yes
**Swaminathan et al. [24]**	*n* = 25 OSA *n* = 13 CPAP	PSG, VF, IOP	CPAP	-	Retrospective cross-sectional study	>2 years	The mean IOP after initiation of CPAP therapy in progressors (14.2 ± 3.3 mmHg) and non-progressors (13.9 ± 2.7 mmHg) was similar (*p* = 0.85) during the course of follow-up (mean: 1.3 years after CPAP initiation).	No
**Ulusoy et al. [36]**	*n* = 106 *n* = 38 CPAP	PSG, IOP, cup/disk ratio	CPAP	*n* = 32 OSA without CPAP, *n* = 36 no OSA	Cross-sectional cohort study	-	IOP and fundus C/D ratio were higher when no CPAP was used (*p* = 0.000), and glaucoma incidence was lower in patients using CPAP (5.2%) in comparison to non-users (12.5%).	Yes

CCT = central corneal thickness; C/D ratio = cup disk ratio; CPAP = continuous positive airway pressure; IOP = intraocular pressure; ML = macular layer; NTG = normal tension glaucoma; OCT = optical coherence tomography; OSA = obstructive sleep apnea; POAG = primary open angle glaucoma; PSD = pattern standard deviation; PSG = polysomnography; RNFL = retinal nerve fiber layer; SAP = standard automated perimetry; VF = visual field; VFI = visual field index.
